# Supplementary material for: The rise of feathered dinosaurs: Kulindadromeus zabaikalicus, the oldest dinosaur with ‘feather-like’ structures
Source: PeerJ. 2019 Feb 1;7:e6239. doi: 10.7717/peerj.6239 (PMC6361000; doi:10.7717/peerj.6239)
Supplement: Supplemental Information 1 — Figure S5 photo credit: Aude Cincotta. [file peerj-07-6239-s001.pdf]

Supplementary Information for: "The rise of feathered dinosaurs: *Kulindadromeus zabaikalicus*, the oldest dinosaur with 'feather-like' structures"

Aude Cincotta [1,2,3], Katerina B. Pestchevitskaya[4], Sofia M. Sinitsa[5], Valentina S. Markevich[6], Vinciane Debaille[7], Svetlana A. Reshetova[5], Irina M. Mashchuk[8], Andrei O. Frolov[8], Axel Gerdes[9], Johan Yans[1], and Pascal Godefroit[2]

[1] Department of Geology, Université de Namur, Namur, Belgium

[2] Directorate 'Earth and History of Life', Royal Belgian Institute of Natural Sciences, Brussels, Belgium

[3] School of Biological, Earth and Environmental Sciences, University College Cork, Cork, Ireland.

[4] Institute of Petroleum Geology and Geophysics. AA Trofimuk, Novosibirsk, Russia

[5] Institute of Natural Resources, Ecology, and Cryology, Chita, Russia

[6] Far East Branch of Russian Academy of Sciences, Vladivostok, Russia

[7] Laboratoire G-Time, Université Libre de Bruxelles, Brussels, Belgium

[8] Institute of Earth's Crust SB RAS, Irkutsk, Russia

[9] Institut für Geowissenschaften, Johann-Wolfgang-Goethe Universiteit, Frankfurt am Main, Germany

Corresponding author:

Aude Cincotta,

Email address: aude.cincotta@ucc.ie

Supplementary Tables

Sample	Quartz (wt %)	K-feldspars (wt %)	Biotite (wt %)	Albite (wt %)	Kaolinite (wt %)	total
Granite 1	38	21.3	9.3	25.7	5.8	100.1
Granite 2	39.4	32	/	28.5	/	99.9
Granite 3	6	59	9.5	21.7	3.4	99.6
Granite 4	15.9	34.7	16.9	32.5	/	100

Supplementary Table S1. X-ray diffraction based compositions of the four igneous rock samples that crop out on top hill. The four samples have a rather similar overall composition (with quartz, feldspars, biotite, and kaolinite), but differ by the relative abundance of their components

	Ratio												Age						
Sample/grain	$^{207}Pb^{a}$ (cps)	U^{b} (ppm)	Pb^{b} (ppm)	$\frac{Th^{b}}{U}$	$^{206}Pb^{c}$ (%)	$\frac{^{206}Pb^{d}}{^{238}U}$	±2s (%)	$\frac{^{207}Pb^{d}}{^{235}U}$	±2s (%)	$\frac{^{207}Pb^{d}}{^{206}Pb}$	±2s (%)	rho[e]	$\frac{^{206}Pb}{^{238}U}$	±2s (Ma)	$\frac{^{207}Pb}{^{235}U}$	±2s (Ma)	$\frac{^{207}Pb}{^{206}Pb}$	±2s (Ma)	conc. (%)
**leucogranite**																			
A06*	78,043	5607	173	0.69	5.3	0.0273	2.2	0.1861	3.4	0.0495	2.7	0.63	173.41	3.7	173	5.5	172	62	101
A07*	72,763	4978	148	0.20	4.8	0.0272	1.9	0.1872	3.3	0.0500	2.7	0.58	172.72	3.2	174	5.3	196	62	88
A08*	11,508	1710	339	31.4	0.4	0.0273	0.8	0.1879	2.0	0.0499	1.9	0.41	173.52	1.4	175	3.3	194	43	90
A09	2600	60	1.6	1.27	2.4	0.0271	1.2	0.1814	2.3	0.0485	2.0	0.50	172.60	2.0	169	3.6	124	47	140
A10	2316	63	1.7	1.43	1.9	0.0271	1.0	0.1844	2.3	0.0493	2.1	0.43	172.54	1.7	172	3.7	163	50	106
A11	2550	87	2.3	1.42	1.1	0.0271	1.1	0.1849	3.0	0.0495	2.8	0.36	172.42	1.8	172	4.7	171	65	101
A12*	24,260	1457	519	62.6	0.0	0.0269	0.8	0.1825	1.7	0.0491	1.5	0.46	171.37	1.3	170	2.7	154	35	111
**T3-7b**																			
A13	2701	111	2.9	1.25	0.6	0.0269	0.9	0.1826	3.7	0.0492	3.6	0.24	171.13	1.5	170	5.8	159	83	108
A14	4712	34	0.9	1.37	13.5	0.0271	2.3	0.1838	4.9	0.0492	4.3	0.48	172.34	4.0	171	7.7	157	100	110
A15	1830	48	1.4	0.61	2.1	0.0293	0.9	0.2026	2.1	0.0500	1.9	0.43	186.48	1.7	187	3.7	199	45	94
A16	1014	26	0.7	1.24	3.2	0.0272	1.2	0.1872	8.1	0.0499	8.0	0.14	172.84	2.0	174	13.1	194	186	89
A17	3604	120	3.2	0.22	0.7	0.0274	1.1	0.1872	2.2	0.0496	1.9	0.51	174.34	1.9	174	3.5	174	44	100
A19	3093	89	2.5	0.93	3.6	0.0293	1.0	0.1991	3.3	0.0494	3.2	0.31	185.85	1.9	184	5.6	166	74	112
A20	4030	57	1.6	1.09	1.2	0.0294	0.8	0.1968	2.5	0.0486	2.4	0.31	186.58	1.5	182	4.2	129	57	145
A21	2752	28	0.8	1.43	3.3	0.0295	0.9	0.2071	3.1	0.0509	2.9	0.30	187.64	1.7	191	5.3	235	67	80
A22	4986	195	5.2	1.24	0.3	0.0273	0.8	0.1855	2.9	0.0493	2.8	0.29	173.67	1.4	173	4.6	161	64	108
A23	1514	61	1.6	1.48	0.4	0.0272	1.0	0.1851	2.2	0.0494	1.9	0.47	172.75	1.7	172	3.4	169	45	102
A24	2279	74	2.1	1.86	1.5	0.0292	1.2	0.1997	2.9	0.0496	2.6	0.43	185.63	2.3	185	4.9	176	60	105
A25	3341	137	3.6	1.35	0.2	0.0270	0.9	0.1844	2.0	0.0495	1.8	0.45	171.99	1.5	172	3.2	170	42	101
A26	1685	53	1.4	1.61	1.2	0.0270	1.2	0.1846	2.4	0.0495	2.1	0.51	171.96	2.1	172	3.9	173	49	99
A27	1905	57	1.5	1.19	1.5	0.0270	1.2	0.1841	2.7	0.0495	2.4	0.44	171.76	2.0	172	4.2	170	56	101
A28	1430	49	1.3	1.15	1.6	0.0272	1.1	0.1836	3.7	0.0491	3.6	0.30	172.71	1.9	171	5.9	151	83	114
A29	2039	76	2.2	1.99	0.5	0.0297	1.0	0.2044	2.3	0.0498	2.1	0.43	189.08	1.8	189	4.0	187	48	101
A30	3865	131	3.8	1.13	0.4	0.0294	0.9	0.2012	2.2	0.0497	2.0	0.43	186.50	1.7	186	3.7	182	46	102
A31	1649	27	0.7	1.20	1.0	0.0279	1.0	0.1902	4.2	0.0494	4.1	0.23	177.59	1.7	177	6.9	168	96	106
A32	1623	29	0.8	1.68	0.3	0.0270	0.9	0.1834	2.2	0.0494	2.0	0.41	171.44	1.5	171	3.5	166	47	103
A39	6094	152	4.3	1.21	4.9	0.0290	0.8	0.1977	4.9	0.0494	4.8	0.16	184.48	1.4	183	8.2	167	113	111
A40	2885	49	1.3	1.52	5.6	0.0273	1.3	0.1868	3.1	0.0496	2.8	0.41	173.60	2.2	174	5.0	179	66	97
A41	2387	40	1.1	1.48	0.5	0.0276	0.9	0.1851	3.5	0.0486	3.4	0.26	175.53	1.6	172	5.6	131	80	134
**T4-3b**																			
A42	6099	251	7.2	1.88	0.0	0.0294	0.8	0.2032	1.7	0.0502	1.6	0.45	186.55	1.4	188	3.0	205	36	91
A43	4124	161	4.6	1.81	0.2	0.0290	0.8	0.1985	1.9	0.0497	1.7	0.41	183.98	1.4	184	3.2	183	40	100
A44	4346	164	4.8	1.11	0.5	0.0298	0.8	0.2052	2.6	0.0499	2.5	0.31	189.26	1.5	190	4.6	194	58	98
A45	1154	49	1.3	1.23	0.3	0.0272	1.0	0.1861	2.0	0.0496	1.7	0.48	173.04	1.6	173	3.2	178	40	97
A46	4039	163	4.4	1.05	0.7	0.0274	0.8	0.1883	3.3	0.0499	3.2	0.24	173.99	1.4	175	5.4	193	75	90
A47	938	30	0.8	0.78	1.7	0.0291	1.5	0.2002	2.7	0.0499	2.2	0.57	184.82	2.8	185	4.5	191	51	97
A48	6697	245	7.0	1.22	0.4	0.0294	0.8	0.1992	2.0	0.0492	1.9	0.38	186.64	1.4	184	3.4	157	44	119
A49	8148	333	8.9	1.22	0.8	0.0272	0.9	0.188	2.1	0.0501	2.0	0.40	173.02	1.5	175	3.5	201	46	86
A50	5682	265	7.0	1.06	0.5	0.0271	0.8	0.1831	1.7	0.0490	1.6	0.43	172.26	1.3	171	2.8	150	37	115
A51	4730	155	4.4	1.30	1.2	0.0289	1.0	0.1971	1.9	0.0494	1.6	0.51	183.82	1.8	183	3.2	168	38	109
BB-16[f]	18900	313	28.4	0.41	0.2	0.0910	1.0	0.7399	1.7	0.05899	1.1	0.59	561	5	562	7	567	24	99.1
Ples[f]	19473	1907	98	0.19	0.3	0.0538	0.8	0.3945	1.0	0.05320	0.5	0.65	338	3	338	3	337	11	100
Mana[f]	48331	1587	3886	95.4	0.3	0.0897	1.9	0.7253	3.2	0.05868	2.8	0.36	554	10	554	14	555	60	100
Itabe[f]	98128	2879	2388	33.3	0.2	0.0822	1.4	0.6493	1.7	0.05724	1.0	0.52	510	7	508	7	501	22	102

[a] within run background-corrected mean ^{207}Pb signal in cps (counts per second).

[b] U and Pb content and Th/U ratio were calculated relative to GJ-1 reference zircon
[c] percentage of the common Pb on the ^{206}Pb. b.d. = below dectection limit.
[d] corrected for background, within-run Pb/U fractionation (in case of $^{206}Pb/^{238}U$) and common Pb using Stacy & Kramers (1975) model Pb composition and subsequently normalised to GJ-1 (ID-TIMS value/measured value); $^{207}Pb/^{235}U$ calculated using $^{207}Pb/^{206}Pb/(^{238}U/^{206}Pb*1/137.88)$
[e] rho is the $^{206}Pb/^{238}U/^{207}Pb/^{235}U$ error correlation coefficient.
[f] Accuracy and reproducibility (2 SD) was checked by repeated analyses (n = 8 to 15) of Plesovice and BB-16 zircon and Itabe and Manangotry monazite; data given as mean with 2 standard deviation uncertainties

Supplementary Table S2. La-ICP-MS data and ages for zircons and monazites (the latter marked with *) collected from Kulinda deposits.

Spores

Annulispora folliculosa (Rogalska) de Jersey, 1959

Biretisporites eneabbaensis Backhouse, 1978

Biretisporites vallatus Sajjadi et Playford, 2002

Cingulatisporites saevus Balme, 1957

Concavisporites junctus (Kara-Mursa) Semenova, 1970

Cyathidites australis Couper, 1953

Cyathidites minor Couper, 1953

Cyathidites punctatus (Delcourt et Sprumont) Delcourt, Dettmann et Hughes, 1963

Densoisporites velatus Weyland et Kreiger, 1953

Dictyophyllidites equiexinus (Couper) Dettmann, 1963

Dictyophyllidites harrisii Couper, 1958

Eboracia granulosa (Tralau) Timochina, 1977

Eboracia torosa (Sachanova et Iljina) Timochina, 1977

Equisetosporites variabilis (Vinogradova) Glushko et Strepetilova, 1994

Gleicheniidites sp.

Leiosphaeridia sp.

Leiotriletes nigrans Naumova, 1953

Leiotriletes pallescens Bolchovitina, 1956

Leiotriletes selectiformis Bolchovitina, 1953

Leiotriletes subtilis Bolchovitina, 1953

Leptolepidites verrucatus Couper, 1953

Neoraistrickia aff. *taylorii* Playford et Dettmann, 1965

Osmunda papillata Bolchovitina, 1953

Osmundacidites jurassicus (Kara-Mursa) Kuzitschkina, 1963

Pilasporites marcidus Balme, 1957

Polycingulatisporites triangularis (Bolchovitina) Playford et Dettmann, 1965

Punctatosporites scabratus (Couper) Norris, 1953

Retitriletes subrotundus (Kara-Mursa) E. Semenova, 1970

Stereisporites bujargiensis (Bolchovitina) Schulz, 1966

Stereisporites compactus (Bolchovitina) Iljina, 1985

Stereisporites congregatus (Bolchovitina) Schulz, 1970

Stereisporites granulatus Tralau, 1968

Stereisporites incertus (Bolchovitina) Semenova, 1970

Stereisporites infragranulatus Schulz, 1970

Stereisporites psilatus (Ross) Pflug, 1953

Stereisporites seebergensis Schulz, 1970

Todisporites major Couper, 1958

Todisporites minor Couper, 1958

Tripartina variabilis Maljavkina, 1949

Undulatisporites fossulatus Singh, 1971

Undulatisporites pflugii Pocock, 1970

Uvaesporites scythicus Semenova, 1970

Verrucosisporites varians Volkheimer, 1971

Pollen

Alisporites bilateralis Rouse, 1959

Alisporites bisaccus Rouse, 1959

Alisporites grandis (Cookson) Dettmann, 1963

Alisporites pergrandis (Bolchovitina) Iljina, 1985

Alisporites similis (Balme) Dettmann, 1963

Araucariacites australis Cookson ex Couper, 1953

Callialasporites dampieri (Balme) Sukh-Dev, 1961

Classopollis classoides Pflug, 1953

Cycadopites dilucidus (Bolchovitina) Ilyina, 1985

Dipterella oblatinoides Maljavkina, 1949

Ginkgocycadophytus sp.

Inaperturapollenites dubius Potonie et Venitz, 1934

Piceapollenites mesophyticus (Bolchovitina) Petrosjanz, 1971

Piceites asiaticus Bolchovitina, 1956

Piceites podocarpoides Bolchovitina, 1956

Pinus divulgata Bolchovitina, 1956

Pinus incrassata Bolchovitina, 1953

Pinus pernobilis Bolchovitina, 1956

Pinus subconcinua Bolchovitina, 1953

Pinus vulgaris (Naumova) Bolchovitina, 1953

Podocarpidites ellipticus Cookson, 1947

Podocarpidites multesimus (Bolchovitina) Pocock, 1970

Podocarpidites rousei Pocock, 1970

Podocarpus major (Maljavkina) Bolchovitina, 1953

Podocarpus paula Bolchovitina, 1956

Podocarpus tricocca (Maljavkina) Bolchovitina, 1953

Protoconiferus funarius (Naumova) Bolchovitina, 1956

Protopicea cerina Bolchovitina, 1956

Protopinus subluteus Bolchovitina, 1956
Pseudopicea grandis (Cookson) Bolchovitina, 1961
Pseudopicea magnifica Bolchovitina, 1956
Pseudopicea monstruosa Bolchovitina, 1956
Pseudopicea variabiliformis Bolchovitina, 1956
Sciadopityspollenites multiverrucosus (Sahanova et Iljina) Iljina, 1985

Supplementary Table S3. List of spore and pollen taxa from the Kulinda locality.

Rock type	Granites				Deposits trench 3						
Sample	1	2	3	4	bb	1F	1C	4	7	10F	10C
SiO_2	69.72	78.36	67.71	68.75	75.93	76.9	68.93	77.8	73.55	57.12	58.37
TiO_2	0.47	0.046	0.44	0.411	0.854	0.731	0.37	0.77	0.376	1.421	0.671
Al_2O_3	15.7	12.44	17.01	15.96	14.6	13.23	16.65	13.6	16.59	22.22	28.85
Fe_2O_3	2.34	0.24	1.91	1.72	<0.01	<0.01	2.88	<0.01	0.13	0.3	0.006
MnO	0.05	0.007	0.031	0.029	0.008	0.004	0.03	0.004	0.004	0.003	0.07
MgO	0.43	0.03	0.32	0.38	0.14	0.11	0.04	0.11	0.05	0.11	0.08
CaO	1.61	0.49	1.44	1.55	0.18	0.11	0.06	0.13	0.14	0.52	0.08
Na_2O	4.12	3.33	4.24	4.3	0.07	0.07	0.28	0.07	0.24	0.03	0.07
K_2O	3.9	5.17	4.9	4.34	1.41	1.62	3.96	1.91	3.23	0.88	1.15
P_2O_5	0.17	<0.01	0.25	0.17	0.05	0.1	0.12	0.11	0.1	3.75	0.15
LOI	1.43	0.19	1.59	1.28	7.02	5.38	5.18	5.36	4.91	10.26	10.62
**Total**	**100.3**	**100.3**	**100.3**	**99.44**	**100.5**	**98.47**	**98.49**	**100.1**	**99.33**	**96.61**	**100.4**
Sc	3	<1	3	3	3	3	5	3	2	8	4
Be	3	5	3	4	4	3	4	4	3	24	5
V	39	<5	38	33	59	34	46	41	26	79	65
Cr	20	<20	20	20	130	90	40	110	80	150	90
Co	4	1	7	3	2	2	7	2	2	1	2
Ni	<20	<20	<20	<20	20	<20	30	<20	30	<20	<20
Cu	10	<10	30	<10	40	40	10	30	50	30	<10
Zn	50	<30	60	40	<30	<30	80	<30	40	30	<30
Ga	21	18	22	22	28	25	18	25	14	40	23
Ge	1.4	2.2	1.7	1.7	2.1	1.9	1.9	2.1	2.1	4.1	1.8
As	23	11	20	18	29	12	149	15	18	24	42
Rb	139	277	156	157	55	60	116	65	95	32	38
Sr	346	37	346	329	345	426	468	382	313	>10,000	584
Y	13.2	2.2	11.6	7.7	20.2	14.8	8.9	20.6	6.6	109	7.9
Zr	407	14	369	338	199	288	241	298	219	142	243
Nb	11.7	2.6	11	10.7	11.7	8.8	7.5	8.7	5.1	20.3	9.8
Mo	<2	<2	<2	<2	5	7	48	4	3	15	9
Ag	1.2	<0.5	1.3	1.3	4.4	0.9	0.8	1	0.9	0.8	0.8
In	<0.1	<0.1	<0.1	<0.1	0.1	<0.1	<0.1	<0.1	0.1	0.1	<0.1
Sn	4	<1	6	2	3	3	1	2	3	4	2
Sb	0.8	0.7	0.8	0.7	5.3	3	6.4	4.1	3.2	14.4	10.4
Cs	6.2	13.8	5.1	4.4	2.4	2.1	1.7	2	1.4	0.9	0.7
Ba	547	64	784	597	304	298	665	322	500	1978	168
Hf	11.6	0.9	11.2	10.4	5.5	9.3	6.6	8.6	6.1	5.9	7.3
Ta	1.43	0.71	1.37	1.51	1.34	0.96	0.94	1	1.51	1.55	0.83
W	2.2	1.7	2.2	2.1	10.4	4.6	3.3	5.4	4.4	11.1	7.2
La	30.2	8.09	31.8	22.4	35.9	36.7	31.1	35.7	19.8	888	27.1
Ce	125	11.8	98.2	76.4	48.3	73.6	65.5	64.6	44.9	1790	47.4
Pr	5.7	1.68	6.03	3.62	6.46	8.02	7.15	6.85	5.35	190	4.83
Nd	19	5.42	19.8	11.2	22.2	28.7	26.5	25.3	21.2	705	17.9
Sm	3.51	0.49	3.69	2.02	3.69	5.09	4.53	4.24	3.73	117	2.9
Eu	0.719	0.143	0.726	0.52	0.925	1.09	0.885	1.05	0.834	23.9	0.748
Gd	2.35	0.33	2.31	1.3	3.21	3.47	3.02	3.79	2.27	73.8	2.19
Tb	0.38	0.06	0.41	0.21	0.5	0.45	0.42	0.57	0.31	8.35	0.3
Dy	2.38	0.38	2.52	1.31	2.8	2.53	2.06	3.2	1.44	32.8	1.53
Ho	0.47	0.08	0.47	0.28	0.56	0.5	0.38	0.63	0.24	4.18	0.27
Er	1.47	0.25	1.35	0.94	1.57	1.38	1.05	1.85	0.66	8.73	0.79
Tm	0.253	0.05	0.254	0.188	0.235	0.234	0.151	0.291	0.096	0.885	0.119
Yb	1.91	0.39	1.76	1.46	1.47	1.54	0.99	1.95	0.64	4.59	0.78
Lu	0.299	0.064	0.283	0.23	0.226	0.243	0.166	0.309	0.106	0.602	0.134
Tl	0.67	1.15	0.68	0.71	<0.05	0.27	0.8	0.28	0.45	0.15	0.19
Pb	23	44	26	26	24	22	34	24	24	542	44
Bi	0.1	0.1	0.1	0.1	<0.1	<0.1	0.3	<0.1	<0.1	0.2	0.3
Th	32.3	11.3	29.5	28.6	8.63	14.2	11.1	14.6	8.73	107	9.56
U	1.97	0.99	2.59	2.21	11.3	32.6	15.7	17	4.94	160	27.1
Th/U	16.4	11.41	11.39	12.94	0.76	0.44	0.71	0.86	1.77	0.67	0.35
∑ REE	193.64	29.23	169.60	122.08	128.05	163.55	143.90	150.33	101.58	3847.84	106.99
LREE/HREE	25.119	21.185	22.113	24.604	15.332	21.379	24.966	15.051	26.131	59.414	24.785
Eu/Eu*	1.176	1.671	1.169	1.508	1.263	1.219	1.124	1.231	1.347	1.209	1.395

Supplementary Table S4. Major (wt %), trace (ppm), and rare earth element (ppm) concentrations of Kulinda deposits. Part I. Samples from the basement and trench 3.

Rock type	Deposits trench 3/3				Deposits trench 4						
Sample	5F	5C	7	8	bb	3F	3C	8	9F	9G	11
SiO_2	79.57	72.67	73.46	72.23	53.46	76.01	65.44	75.01	78.72	72.29	68.55
TiO_2	0.745	0.384	0.896	0.585	0.588	0.884	0.749	0.314	0.734	0.447	0.788
Al_2O_3	12.37	18.99	16.17	17.01	10.76	13.28	20.51	14.54	12.3	17.59	16.76
Fe_2O_3	<0.01	0.11	0.25	0.13	21.1	0.01	1.34	1.25	<0.01	<0.01	0.16
MnO	0.009	0.005	0.004	0.01	0.655	0.006	0.05	0.063	0.005	0.006	0.013
MgO	0.11	0.05	0.17	0.09	0.41	0.19	0.06	0.04	0.13	0.08	0.31
CaO	0.15	0.08	0.16	0.09	0.29	0.21	0.47	0.15	0.19	0.16	0.35
Na_2O	0.07	0.13	0.05	0.14	0.08	0.09	0.08	0.31	0.08	0.14	0.08
K_2O	1.37	1.98	1.46	2.22	2.42	1.51	2.92	4.38	2.19	3.16	2.81
P_2O_5	0.13	0.08	0.11	0.12	0.32	0.06	0.33	0.1	0.12	0.12	0.18
LOI	6.02	6.27	7.31	6.32	9.16	6.65	7	3.87	5.63	5.94	8.99
**Total**	**100.8**	**100.8**	**100.1**	**99.4**	**100.3**	**99.57**	**99.28**	**100**	**100.4**	**100.3**	**99.45**
Sc	4	2	5	3	18	3	5	4	5	3	5
Be	4	4	5	4	7	4	3	3	3	3	7
V	51	27	63	45	147	60	52	34	58	40	67
Cr	100	90	110	80	60	90	40	80	60	40	80
Co	3	2	1	4	9	3	33	10	2	3	7
Ni	<20	<20	30	20	40	<20	50	40	<20	<20	40
Cu	40	<10	30	10	20	30	20	40	30	20	40
Zn	<30	<30	<30	40	180	40	150	60	<30	<30	60
Ga	23	12	20	20	16	27	22	15	21	15	22
Ge	1.5	1.7	1.8	1.9	1.8	1.3	1.2	1.9	1.3	1.3	1.6
As	29	17	21	17	94	62	105	63	45	38	41
Rb	55	62	60	69	95	63	105	119	84	99	115
Sr	662	464	483	569	710	141	200	490	432	341	575
Y	9.1	4.1	11.2	8.1	37.9	7.5	12.1	7.1	9.5	5.6	14.7
Zr	190	161	181	186	201	127	265	137	202	234	207
Nb	8.8	5.8	9.3	6.7	9.9	9.2	8.4	4.8	10	6	9.2
Mo	3	3	<2	5	11	7	11	23	5	6	3
Ag	3.3	0.6	0.6	0.6	0.9	6.7	0.9	0.7	5.6	1	0.8
In	<0.1	0.1	0.1	<0.1	0.1	<0.1	<0.1	<0.1	<0.1	0.1	0.1
Sn	3	1	2	2	2	2	2	3	3	2	3
Sb	4	2.5	2.1	1.9	2.2	5.6	4.2	4.4	5.2	3.1	1.9
Cs	3.1	1.2	3.2	1.6	3	5	0.9	1.5	3	1.6	4.8
Ba	273	271	264	413	389	249	160	758	293	346	409
Hf	5.5	5.8	4.9	4.9	5.6	3.9	8	4.3	5.4	6.8	6.5
Ta	1.28	0.58	1.04	0.75	0.83	1.08	0.67	0.59	1.2	0.63	0.97
W	7.8	3.7	5.8	4.2	4.3	10.2	4.8	3.8	8.8	3.7	5
La	43.4	22.2	34.3	35.9	50.2	14.9	30.1	30	39.3	27.3	53.5
Ce	88.5	44.7	69.4	70.3	138	24.3	53.4	54.1	67.5	46.8	112
Pr	9.43	4.84	7.44	7.32	17.1	2.3	5.4	6.04	6.74	4.52	12.1
Nd	34	17.3	26.8	26.9	70.1	7.14	19.2	21.8	22.3	15.6	43.7
Sm	5.88	3.17	4.69	4.79	12.1	1.03	3.31	3.32	3.23	2.2	8.4
Eu	1.36	0.794	0.989	0.986	2.39	0.36	0.879	0.808	0.831	0.587	1.55
Gd	4.26	2.17	3.13	3.13	9.97	0.8	2.28	2.22	2.11	1.42	5.28
Tb	0.49	0.27	0.4	0.39	1.28	0.13	0.36	0.33	0.27	0.19	0.69
Dy	2.15	1.12	1.9	1.79	6.73	0.88	2.24	1.74	1.47	1.09	3.2
Ho	0.31	0.16	0.36	0.29	1.22	0.18	0.44	0.28	0.28	0.21	0.5
Er	0.83	0.41	1.07	0.78	3.32	0.59	1.21	0.72	0.82	0.57	1.34
Tm	0.113	0.061	0.171	0.121	0.47	0.1	0.165	0.096	0.129	0.09	0.201
Yb	0.8	0.47	1.15	0.84	2.91	0.7	1.01	0.62	0.94	0.68	1.34
Lu	0.149	0.083	0.163	0.124	0.442	0.121	0.145	0.105	0.158	0.111	0.203
Tl	<0.05	0.24	0.25	0.26	0.46	<0.05	0.52	1.07	<0.05	0.33	0.46
Pb	23	15	28	18	51	24	29	29	39	24	27
Bi	<0.1	<0.1	<0.1	<0.1	0.4	<0.1	0.3	0.2	<0.1	<0.1	<0.1
Th	16.2	9.52	14.1	15	13.8	5.75	7.91	7.53	14.5	8.96	21
U	22.3	3.28	16.3	11.1	40.3	10.8	16.7	11.7	9.81	9.96	46
Th/U	0.73	2.90	0.87	1.35	0.34	0.53	0.47	0.64	1.48	0.90	0.46
∑ REE	191.67	97.75	151.96	153.66	316.23	53.53	120.14	122.18	146.08	101.37	244.00
LREE/HREE	36.210	34.592	26.456	32.392	16.821	18.008	19.406	28.769	33.401	32.037	29.609
Eu/Eu*	1.277	1.423	1.213	1.197	1.023	1.864	1.504	1.399	1.496	1.561	1.094

Supplementary Table S4. Major (wt %), trace (ppm), and rare earth element (ppm) concentrations of Kulinda deposits. Part II. Samples from the trenches 3/3 and 4.

Supplementary Figures

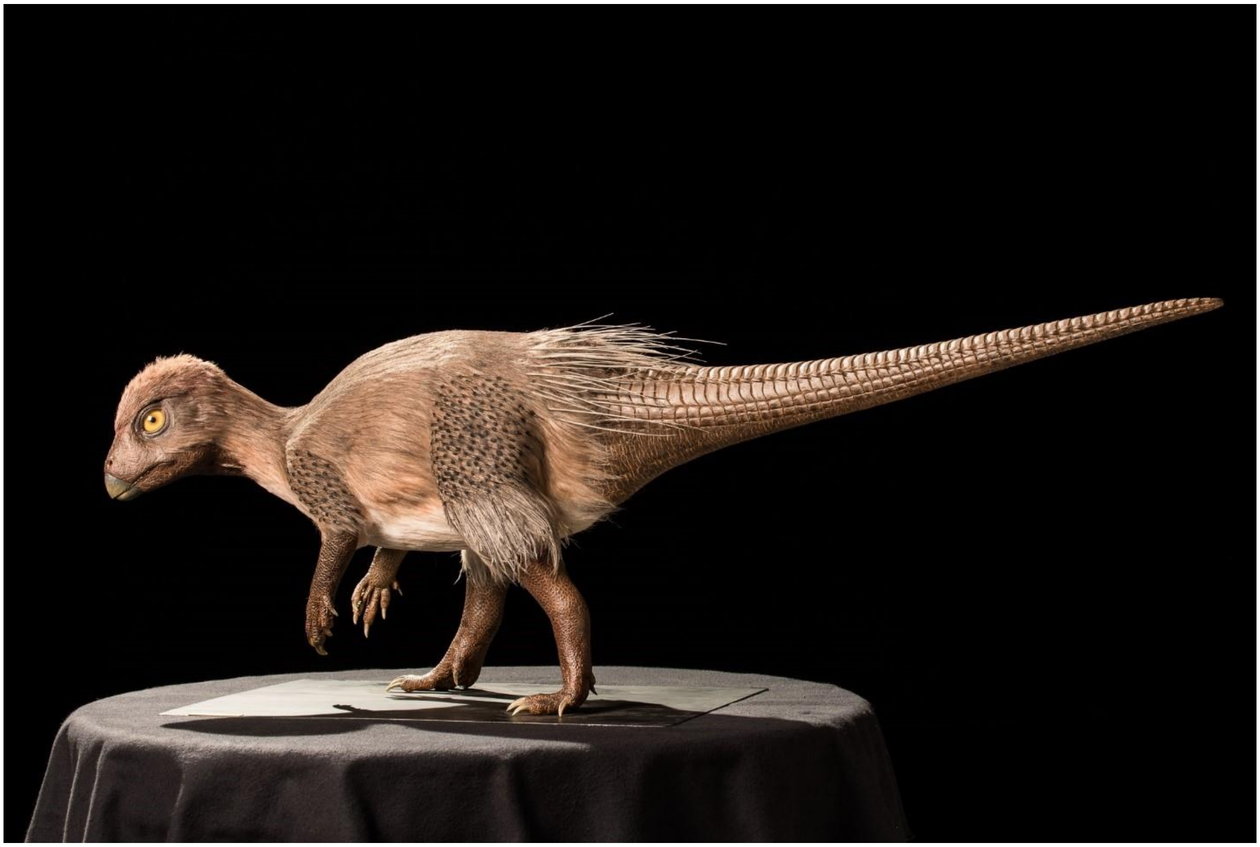

Supplementary Figure S1. 3D reconstruction of *Kulindadromeus zabaikalicus*. The neornithischian dinosaur is reconstructed with its integumentary coverage consisting of epidermal, overlapping scales on the tail and the distal part of the tibiae, non-overlapping scales around the manus, tarsus, metatarsus, and pes, monofilaments around the thorax, on the back and skull, clusters of filaments and their basal plate on the humerus and femur, and ribbon-shaped structures on the proximal part of the tibiae. 3D reconstruction: J. Dos Remedios and M. Mohamed. Photograph credit: RBINS (T. Hubin).

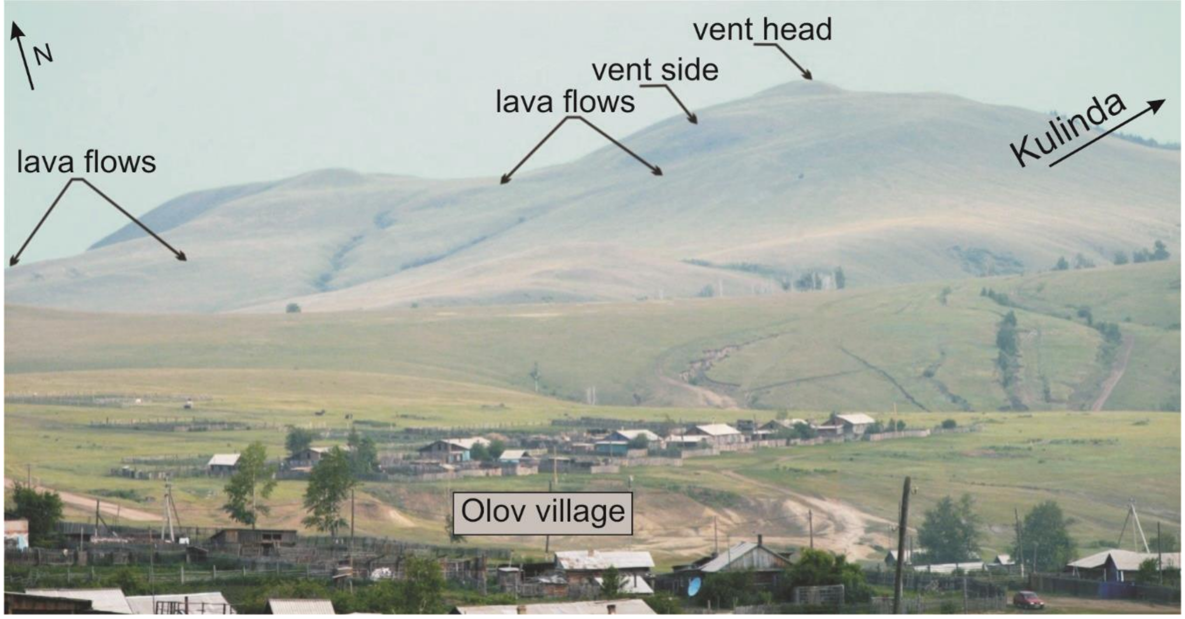

Supplementary Figure S2. Photograph of the Pharaoh volcano located in the background of the Olov village, southwest of Kulinda locality (after Kozlov, 2011). Volcanic rocks (trachyandesites) were observed on the western part of the edifice (see the arrows). Radiochronological dating indicates an Early Cretaceous age for these deposits (Kozlov, 2011).

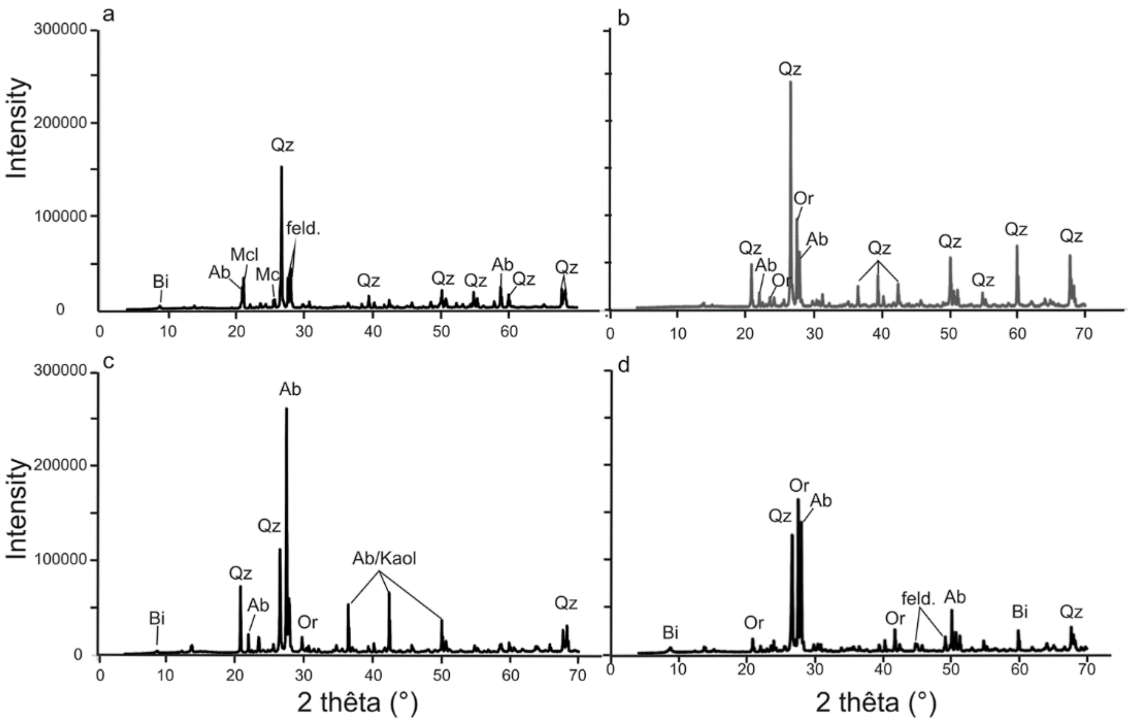

Supplementary Figure S3. X-ray diffraction spectra of igneous rock samples from Kulinda. The spectra show the main mineralogical features for four igneous rock samples that crop out on top hill: (a) biotite granite, (b) granite, (c) and (d) quartz-biotite monzonites. All samples contain quartz and feldspars in various, generally high, abundances, together with, less, biotite and kaolinite. The percentages of the main mineral phases are shown in Supplementary Table S1. Abbreviations: Qz, quartz; Ab, albite; Or, orthoclase; Mcl, microcline; feld., feldspars, refer to probable solid solution between albite and orthoclase; Bi, biotite; Kaol., kaolinite.

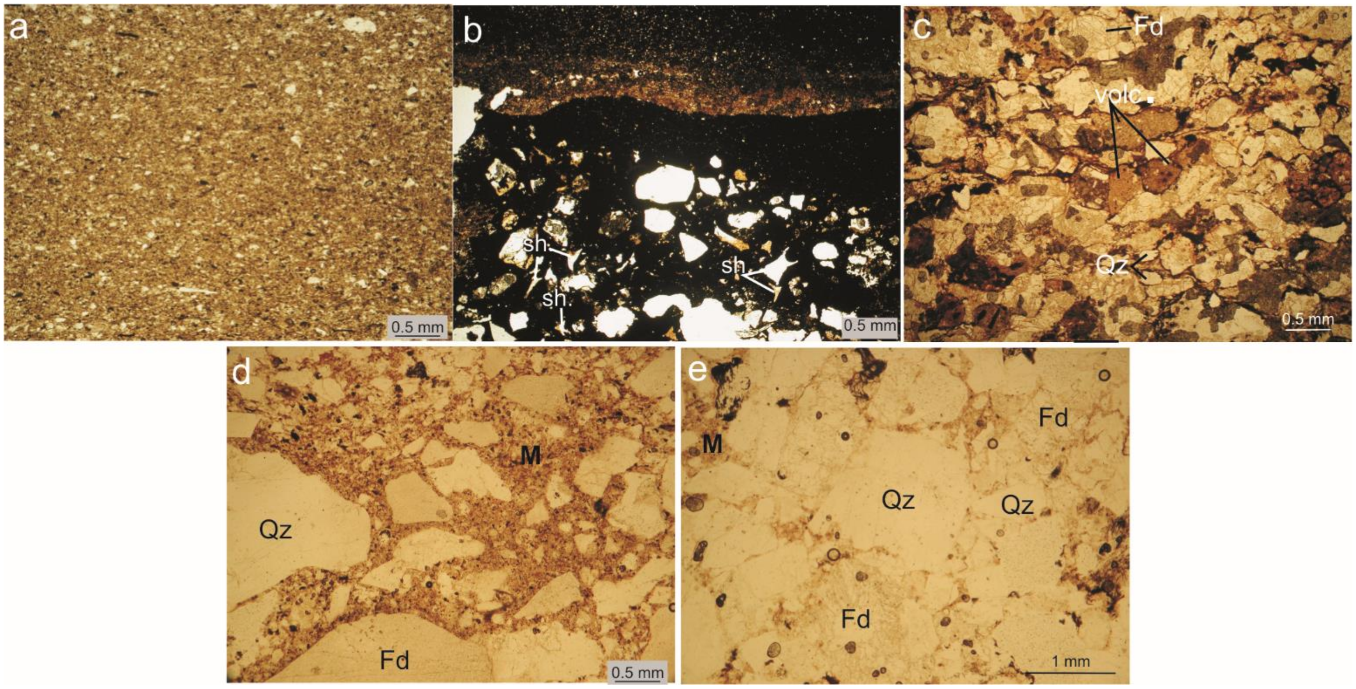

Supplementary Figure S4. Microfacies encountered in Kulinda deposits. (a) mudstone from trench 3 (sample T3-3), (b) tuffaceous siltstone from trench 4 (sample T4-5), (c) lithic arenite from trench 4 (sample T4-5), (d) greywacke from trench T3/3 (sample T3/3-5), and (e) brecciated sandstone from trench 3 (sample T3-7). Abbreviations: Qz, quartz; volc, volcanic lithics; sh, glass shards; Fd, feldspars; M, matrix.

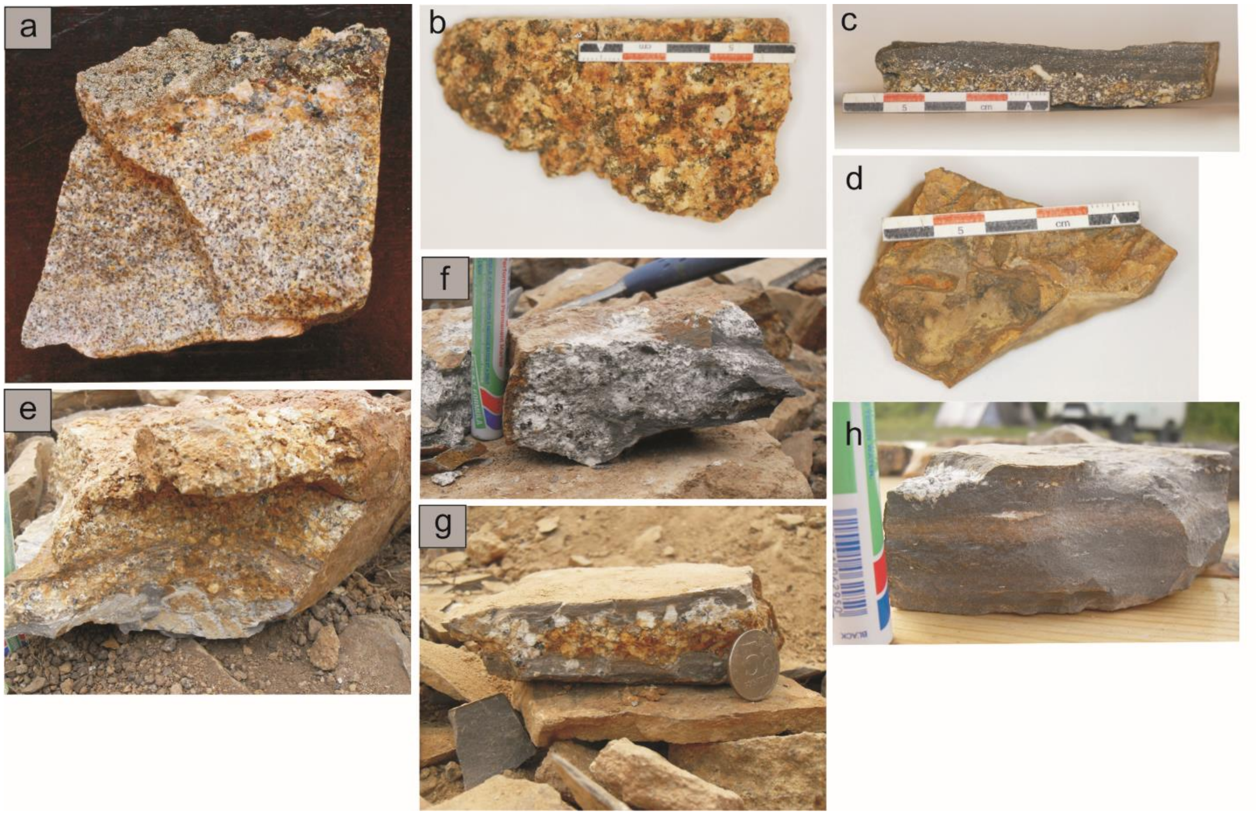

Supplementary Figure S5. Selected rock samples from Kulinda locality. The photographs show (a) a granite and (b) a biotite granite collected on top hill, (c) and (d) greywackes (siltstones) from trench 4, (e) and (f) breccia and brecciated sandstone from trench 3, (g) very coarse-grained sandstone and (h) mudstone from trench 3/3.

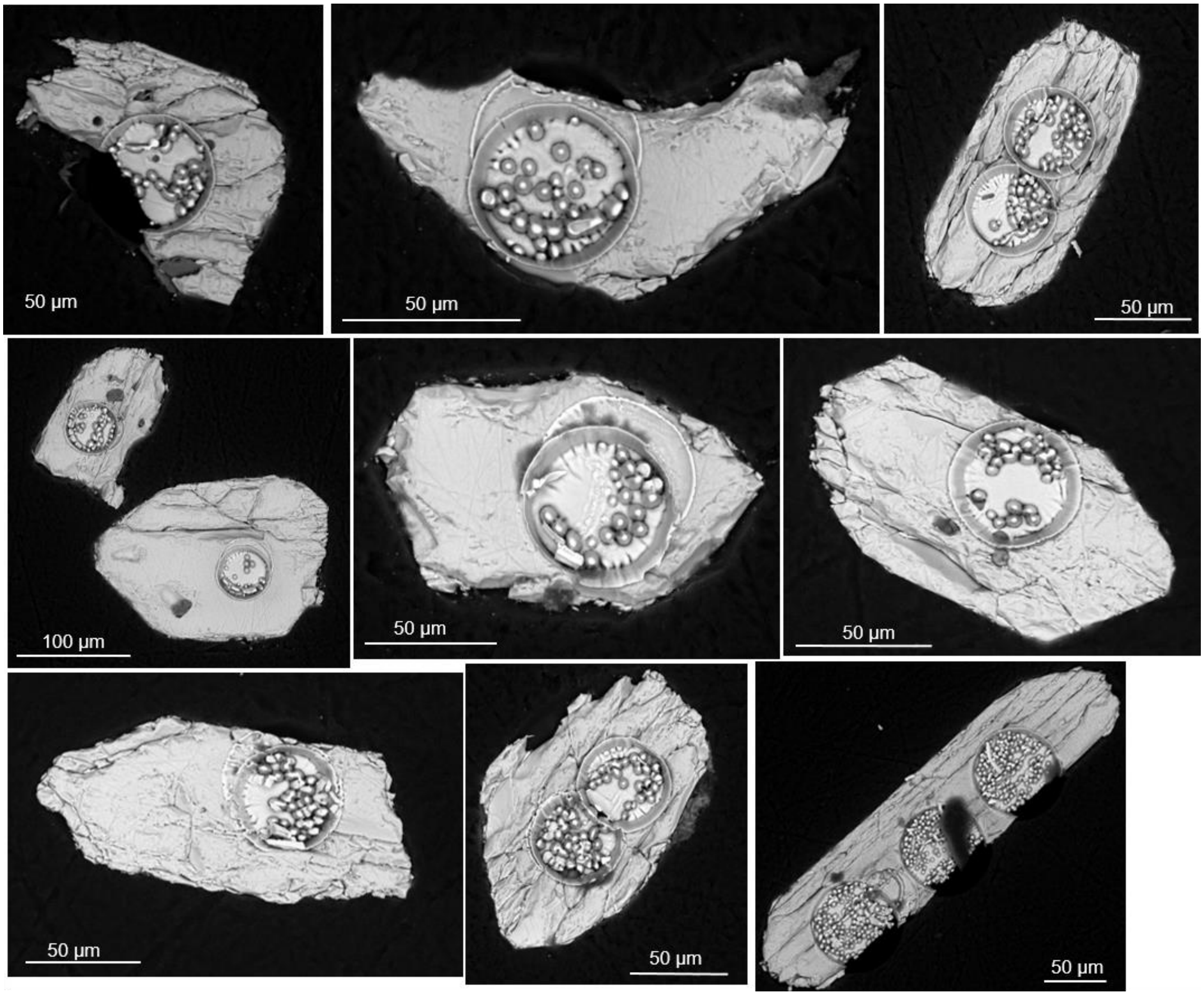

Supplementary Figure S6. Backscattered electron (BSE) images of selected zircons from the volcaniclastic sediments. These images show the location of the laser beam used for radiometric (LA-ICP-MS) analyses of the minerals.

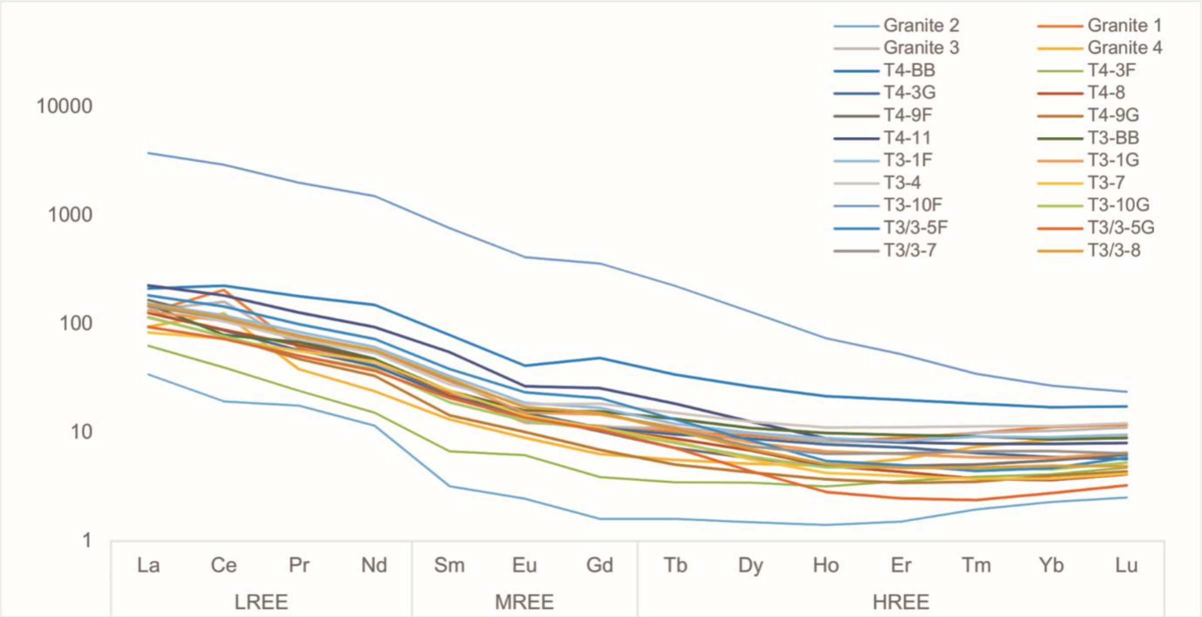

Supplementary Figure S7. Rare earth element (REE) chondrite-normalized diagram for Kulinda deposits. The distribution pattern is rather similar for all samples and show an enrichment in light REE (LREE) with respect to the heavy REE (HREE). Values for normalization were taken from Sun & McDonough (1989).

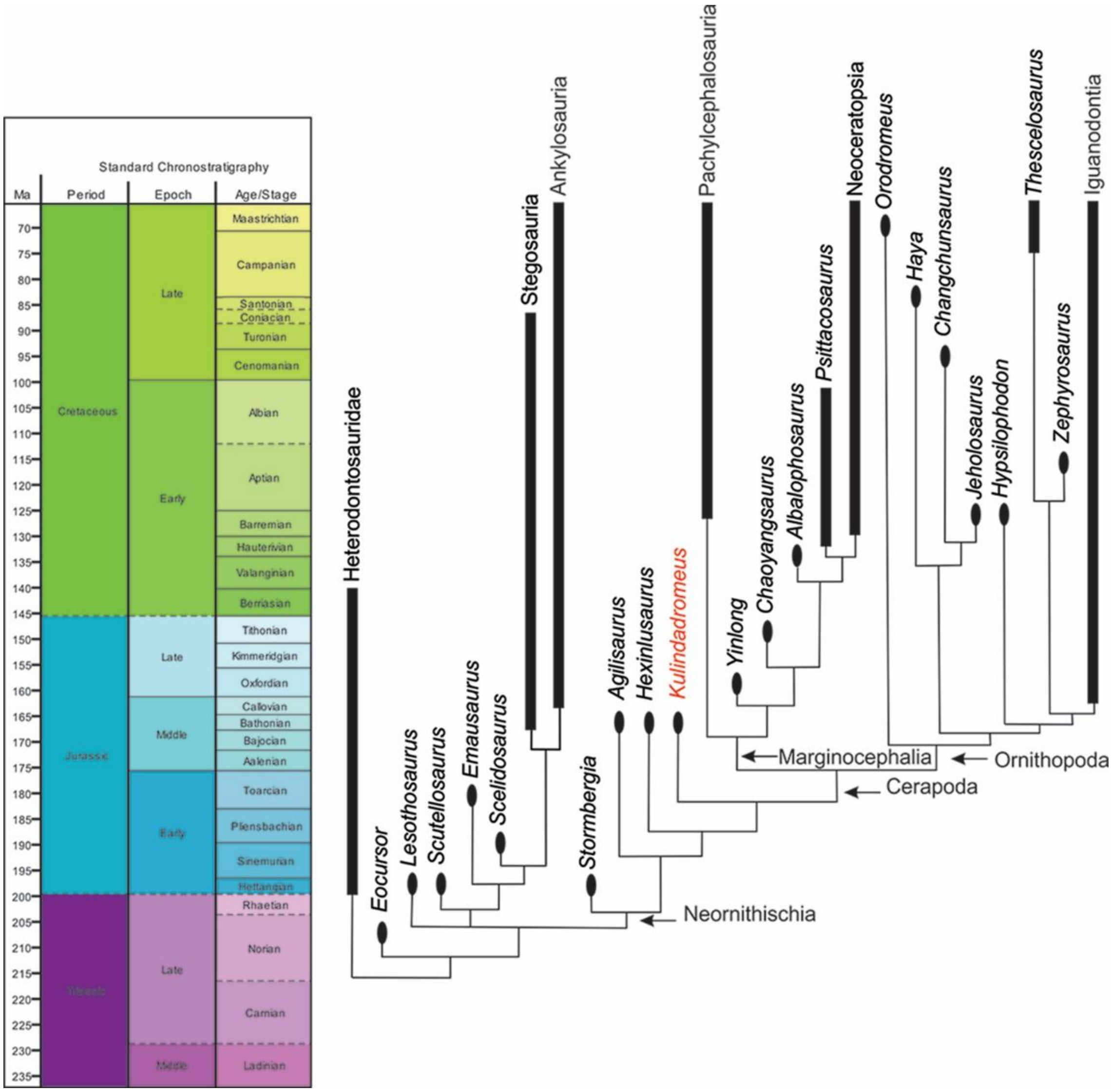

Supplementary Figure S8. Time-calibrated phylogeny of basal ornithischian dinosaurs (modified from Godefroit et al., 2014).

<u>**Supplementary Methods**</u>

Composition and provenance of the deposits. Kulinda locality is located in the Chernyshevsky District of the Transbaikal Region, in south-eastern Siberia (Russia). During the last field campaign conducted in the summer of 2015, two trenches (T4 and T3) - previously excavated on a steep hillside in 2013 (Godefroit et al., 2014) - have been studied, along with a new third trench (T3/3) located at an intermediate elevation, between the trenches T4 and T3 (Fig. 3 in the main manuscript). The geochemistry of the Kulinda deposits have been studied in detail using a total of thirty-six rock samples. Twelve samples have been collected from trench 4, nine from trench 3/3, and eleven from trench 3. In addition, four samples were collected from the plutonic intrusion. Twenty-two samples (seven from trench 4, four from trench 3/3, and seven from trench 3) were analysed by fusion ICP-MS for major and trace element analyses. For this purpose, samples have been crushed to a grain size <125 µm. Ten major elements - Si, Al, Fe, Mn, Mg, Ca, Na, K, Ti, and P -, thirty one trace elements - Sc, Be, V, Cr, Co, Ni, Cu, Zn, Ga, Ge, As, Rb, Sr, Y, Zr, Nb, Mo, Ag, In, Sn, Sb, Cs, Ba, Hf, Ta, W, Tl, Pb, Bi, Th, U -, and the rare earth elements - La, Ce, Pr, Nd, Sm, Eu, Gd, Tb, Dy, Ho, Er, Tm, Yb, Lu - were analysed. Total iron was expressed as Fe_2O_3 (total). Values for major elements and rare earth elements have been normalized to chondrites (McDonough & Sun, 1995), and trace elements have been normalized to the Upper Continental Crust (UCC; McLennan, 2001).

The mineralogy of selected rock samples from Kulinda has been studied by X-ray diffraction (XRD) analysis using a PANanalytical Empyrean diffractometer (at the Royal Belgian Institute of Natural Sciences) with Cu K_α radiation. A tube voltage of 45 kV and a tube current of 40 mA were used. The goniometer scanned from 3° to 69° 2θ for the bulk rock. The semi-quantitative interpretation of data was made using Visual Crystal 6 software.

Backscattered electron (BSE) imaging of the zircons was performed using an environmental QUANTA 200 (FEI) scanning electron microscope at the Royal Belgian Institute of Natural Sciences. The mean acceleration voltage is 20 kV.

Supplementary Text

Geological setting. Kulinda (Transbaikal region, south-eastern Siberia, Russia; GPS coordinates: 52°31'N; 116°42'E) is located at the boundary between the Onon Island Arc and the active margin of Siberia, close to the eastern part of the Mongolia-Okhotsk suture (Xu et al., 1997; Zorin, 1999; Kravchinsky et al., 2002). The Mongol-Okhotsk Orogenic Belt forms the eastern and youngest segment of a major geotectonic unit, the Central Asian Orogenic Belt (Wang et al., 2015) and is related to the suture of the Mongol-Okhotsk Ocean, a huge segment of the paleo-Pacific (Zorin et al., 1995), located between Siberia, the Onon Island Arc, and the Mongolia-North China continent in the Devonian time (Zorin et al., 2001; Donskaya et al., 2013; Wang et al., 2015). The subduction of the oceanic lithosphere beneath Siberia and Mongolia from the Early Permian led to the scissor-like eastward collision of Mongolia-North China and Siberia continental blocks (Xu et al., 1997; Zorin, 1999). The collision between the two blocks lasted since the Late Permian up to the Early Jurassic (Zorin, 1999; Donskaya et al., 2013). The exact closure time of the ocean is still debated, but it likely occurred during the Late Jurassic-Early Cretaceous (Zorin et al., 2001; Kravchinsky et al., 2002; Jolivet et al., 2013; Vorontsov, Yarmolyuk & Komaritsyna, 2016). However, the closure of the Mongol-Okhotsk Ocean in south-eastern Siberia likely took place at the Early-Middle Jurassic boundary (Zorin, 1999; Zorin et al., 2001), or during the early Middle Jurassic (Jolivet et al., 2013).

Intense magmatism occurred during the Permian, Triassic and Early Jurassic in south-eastern Siberia, because of the subduction of the Mongol-Okhotsk oceanic plate beneath Siberia (Zorin, 1999; Zorin et al., 2001). Granite and granodiorite plutons are therefore widespread in the region, with calc-alkaline rock suites located in the front part of the active continental margin, and sub-alkaline suites in the back part of the continental margin (Tomurtogoo et al., 2005). Although magmatism dropped in the Jurassic period, probably related to the ending of the subduction (Donskaya et al., 2013), Middle-Late Jurassic granitoids and other various igneous rocks are known in the region of Kulinda (Zorin et al., 2001). Marine sedimentation occurred in central and south-eastern Transbaikalia until the early Middle Jurassic, followed by a period of continental rifting reflected by the formation of grabens in the region (Starchenko, 2010; Rudenko & Starchenko, 2010). Later magmatic events in the region occurred in the Late Jurassic-Early Cretaceous and are the consequence of the post-collisional rifting (Zorin et al., 1995; Zorin, 1999; Stupak, Kudryashova & Lebedev, 2016).

Discussion on the composition of the Kulinda dinosaur fauna. All the ornithischian material discovered at Kulinda belongs to the basal neornithischian *Kulindadromeus zabaikalicus* (Godefroit et al., 2014). An alternative interpretation for the dinosaur fauna at Kulinda was proposed (Alifanov & Saveliev, 2014; 2015) and named three new taxa from this locality: the 'hypsilophodontian' ornithopods *Kulindapteryx ukureica* and *Daurosaurus olovus* (Alifanov & Saveliev, 2014), and the 'nqwebasaurid' ornithomimosaur *Lepidocheirosaurus natalis* (Alifanov & Saveliev, 2015). Detailed description of the osteology of the dinosaurs from Kulinda is beyond the scope of this paper, but some brief comments are made below.

It should be noted that these interpretations were not done with the modern taxonomic standards for elaborating the classification schemes: they do not use cladistic methods for inferring the

phylogenetic relationships between taxa. *Kulindapteryx* and *Daurosaurus* only differ in the structure of their ischia (the only bone that is preserved in both taxa), but those differences can easily be explained by differences in the orientation of the bones: PIN 5434/25a, the holotype of *Kulindapteryx ukureica*, is clearly exposed in dorsal view, not in lateral view as hypothesized by the authors. We therefore consider *Kulindapteryx ukureica* and *Daurosaurus olovus* as nomina dubia, and that the specimens both fall within the *Kulidadromeus zabaikalicus* hypodigm.

In their paper, the authors claimed that the caudal vertebrae and associated scales referred to *Kulindadromeus zabaikalicus* (Godefroit et al., 2014) belong to a new ornithomimosaur, *Lepidocheirosaurus natalis* (Alifanov & Saveliev, 2015). This interpretation is based on analysis of one partially articulated manus and of caudal vertebrae associated by caudal scales. The caudal vertebrae show a spool-shaped centrum, well-developed postzygapophyses and weakly developed neural spine; these features were thought to be characteristic of theropods and to contrast with the vertebrae of bipedal Ornithischia (Alifanov & Saveliev, 2015), characterized by better developed neural spines, a cylindrical centrum and weakly developed postzygapophyses. However, this interpretation is apparently based on direct comparisons with the ornithopod *Hypsilophodon foxii* and lacks a broader phylogenetic context (Alifanov & Saveliev, 2015). For example, caudal vertebrae of more basal ornithischians, e.g., the heterodontosaurid *Tianyulong confuciusi* (see Sereno, 2012, fig.25), closely resemble those discovered at Kulinda: from about the tenth vertebra, the centrum is elongated and spool-shaped in lateral view, the neural spines are reduced to a ridge, and both the pre-and postzygapophyses are long, extending beyond the level of the articular surfaces of the centra. Except for the absence of evidence for ossified tendons, the caudal structure in *Tianyulong* is remarkably similar to that in dromaeosaurid theropods (Sereno, 2012). Furthermore, the hand of *Lepidocheirosaurus natalis* from Kulinda closely resembles that

of *Tianyulong* (see Sereno, 2012, fig. 27). Combined, these observations strongly indicate a lack of support for the hypothesis that basal ornithomimosaurs were present at Kulinda and that the caudal and manus material described by (Alifanov & Saveliev, 2015) can be confidently attributed to basal ornithischians, such as *Kulindadromeus zabaikalicus*. The most parsimonious interpretation of the Kulinda bone beds is thus that they represent accumulation of a monospecific dinosaur assemblage (Godefroit et al., 2014).

Paleofloral assemblage. The macrofloral assemblage recovered from Kulinda deposits is relatively poor and mostly consists of fossil plants that are widespread in strata from the Siberian paleofloristic region. Moss sprouts are rare and their remains are most commonly subordinate in the deposits. They nonetheless comprise remains of the dichotomous mosses *Hepaticites cf. arcuatus* (L. *et* H.) Harris and the cormophytic mosses *Muscites samchakianus* Srebr. Gymnosperms are mainly represented by Coniferales – *Elatides ovalis* Heer, *Pityospermum* sp., *Carpolithes*, the archaic Pinaceae *Schizolepidopsis* −, and Leptostrobales – *Czekanowskia* − trees and shrubs. Horsetails (*Equisetites*), ferns (*Coniopteris*), conifers (*Pityophyllum*), and leptostrobaleans are represented by highly fragmented specimens identifiable at the generic level, only. In trench 4, one can identify the following taxa: *Algites* sp., *Hepaticites* cf. *arcuatus*, *Equisetites* sp., *Czekanowskia* ex. gr. *rigida*, *Elatides ovalis*, and *Schizolepidopsis elegans*; in trench 3/3: *Hepaticites* cf. *arcuatus*, *Carpolithes sp*., and *Algites sp*.; and in trench 3: *Hepaticites* cf. *arcuatus*, *Muscites samchakianus*, *Schizolepidopsis* sp., and *S. moellerii*.

The assemblage is characterized by the predominance of small herbaceous plants represented by the mosses *Hepaticites* cf. *arcuatus* and *Muscites samchakianus*. Such delicate moss stems are generally rare in Jurassic deposits, and their remains are, commonly, not abundant in floral oryctocoenoses (Vakhrameev, 1991). Nevertheless, mosses are widespread in all regions of the

world since the Palaeozoic, and some species can occupy harsh environments (Bardunov, 1984). It should be noted that, at Kulinda, ferns, arthrophytes, and leptostrobaleans are only represented by highly fragmented remains and their identification was possible at the generic level, only. Gymnosperms are abundant in some layers, and especially Coniferales and Leptostrobales, which formed trees and bushes. *Czekanowskia* ex gr. *rigida* is a typical Siberian gymnosperm species characterized by a wide stratigraphic extension: it is known from the Lower Jurassic to the Upper Jurassic (Vakhrameev, 1991; Samylina & Kiritchkova, 1993). The moss *Hepaticites arcuatus* was originally found in the Middle Jurassic of Yorkshire (Harris, 1961; 1964). The absence of epidermal structure in the specimens from Kulinda locality allows the definition of this species in the open nomenclature, only. *Elatides ovalis*, *Schizolepidopsis moelleri*, *S. elegans*, *S. levis* are also interpreted as Early-Middle Jurassic species (Vakhrameev, 1970; 1991; Mogutcheva, 2009). The modern morphology of the conifers was already established in the early Mesozoic (Andrews, 1961). The evolution of the axillary region led to the formation of seeds with spliced scales, which is a typical feature for the group and particularly for the genus *Schizolepidopsis*. The first occurrences of *Schizolepidopsis* are reported from the Triassic of the Fergana Valley in eastern Uzbekistan (Stiksel, 1966). All specimens identified at Kulinda were observed in the Lower Jurassic of Central Asia as well (Turutonova-Ketova, 1950). However, they seem more abundant in the Ukurey Formation, where the presence of unique morphotypes might reflect endemicity. Abundant bifurcate seed scales of *Schizolepidopsis* are typical for the Middle and Upper Jurassic deposits of Siberia (Vakhrameev, 1991). The fern *Coniopteris* appeared in the late Early Jurassic but became common since the Middle Jurassic (Vakhrameev, 1991; Deng & Lu, 2006). *Muscites samchakianus* is reported in Middle-Upper Jurassic deposits from Transbaikal region (Srebrodolskaya, 1980). The taxonomic composition of the paleofloral assemblages from Kulinda

locality cannot therefore give a more detailed age than the Middle Jurassic-Early Cretaceous time range for the deposits.

Supplementary references

Andrews HN. 1961. *Studies in paleobotany*. (Wiley).

Bardunov LV. 1984. *Ancient flora on the land [Drevneyshie na sushe]*. (Nauka).

Deng S & Lu Y. 2006. The Mesozoic Dicksoniaceous ferns: characteristics, distribution, origin and evolutionary trends. *Global Plant Letters* 1:9-29.

Donskaya TV, Gladkochub DP, Mazukabzov AM & Ivanov AV. 2013. Late Paleozoic – Mesozoic subduction-related magmatism at the southern margin of the Siberian continent and the 150 million - year history of the Mongol-Okhotsk Ocean. *Journal of Asian Earth Sciences* 62:79-97. DOI: 10.1016/j.jseaes.2012.07.023.

Harris TM. 1961. The Yorkshire Jurassic Flora. I. Thallophyta-Pteriodophyta. Brit. Mus. *Nat. Hist.), London. 212pp. Pteridophyta, Palaeo (PMBD, 185305195).*

Harris TM. 1964. *The Yorkshire Jurassic Flora. II. Caytoniales, Cycadales & Pteridosperms*. (British Museum (Natural History).

Kravchinsky VA, Cogné JP, Harbert WP & Kuzmin MI. 2002. Evolution of the Mongol-Okhotsk Ocean as constrained by new palaeomagnetic data from the Mongol-Okhotsk suture zone, Siberia. *Geophysical Journal International* 148:34-57.

McDonough WF & Sun SS. 1995. The composition of the Earth. *Chemical Geology* 120(3-4):223-253. DOI: 10.1016/0009-2541(94)00140-4.

McLennan SM. 2001. Relationships between the trace element composition of sedimentary rocks and upper continental crust. *Geochemistry, Geophysics, Geosystems* 2(4) DOI:10.1029/2000GC000109.

Mogutcheva NK. 2009. Problems of phytostratigraphy and the correlation of the Lower Jurassic continental sediments in West Siberia and Kuznetsk and Kansk-Achinsk basins. *Stratigraphy and Geological Correlation* 17:283-290. DOI: 10.1134/S0869593809030046.

Samylina VA. & Kiritchkova AI. 1993. The genus Czekanowskia Heer: principles of systematics, range in space and time. *Review of palaeobotany and palynology* 79:271-284. DOI: 10.1016/0034-6667(93)90026-Q.

Sereno PC. 2012. Taxonomy, morphology, masticatory function and phylogeny of heterodontosaurid dinosaurs. *ZooKeys* 226(1).

Stacey JT & Kramers J. 1975. Approximation of terrestrial lead isotope evolution by a two-stage model. *Earth and planetary science letters* 26:207-221.

Srebrodolskaya IN. 1980. New Late Mesozoic bryopsids from Transbaikalia [Novye pozdnemezozoiskie listostebel'nye mkhi iz Zabaikal'ya]. *Proceedings of VSEGEI (Vsesoyuznogo Nauchno-Issledovatelskogo Geol. Inst.)* 204:27-28.

Sikstel TA. 1966. *Permian phytostratigraphy of Middle Asia [K fitostratigrafii permi v Sredney Azii]*. (Tashkent: Fan).

Stupak FM, Kudryashova EA & Lebedev VA. 2016. On the Jurassic volcanism and on volcanoes in the Shadoron Basin, eastern Transbaikalia. *Journal of Volcanology and Seismology* 10: 18-31. DOI: 10.1134/S0742046316020056.

Sun SS & McDonough WF. 1989. Chemical and isotopic systematics of oceanic basalts: implications for mantle composition and processes. *Geological Society, London, Special Publications* 42:313-345. DOI: 10.1144/GSL.SP.1989.042.01.19.

Turutanova-Ketova. 1950. In: *Palaeontological questions [Voprosy paleontologii]* 237-347 (Leningrad University).
Vakhrameev VA. 1970. Range and palaeoecology of Mesozoic conifers, the Cheirolepidiaceae. *Paleontological Journal* 4:12-24.
Vakhrameev VA. 1991. In *Jurassic and Cretaceous floras and climates of the Earth*, Cambridge University Press.
Vorontsov AA, Yarmolyuk VV & Komaritsyna TY. 2016. Late Mesozoic – Early Cenozoic rifting magmatism in the Uda sector of Western Transbaikalia. *Russian Geology and Geophysics* 57:723-744 DOI:10.1016/j.rgg.2015.09.015.
Wang T, Guo L, Zhang L, Yang Q, Zhang J, Tong Y & Ye K. 2015. Timing and evolution of Jurassic–Cretaceous granitoid magmatisms in the Mongol–Okhotsk belt and adjacent areas, NE Asia: Implications for transition from contractional crustal thickening to extensional thinning and geodynamic settings. *Journal of Asian Earth Sciences* 97:365-392. DOI: 10.1016/j.jseaes.2014.10.005.
Xu X, Harbert W, Dril S & Kravchinsky V. 1997. New paleomagnetic data from the Mongol-Okhotsk collision zone, Chita region, south-central Russia: implications for Paleozoic paleogeography of the Mongol-Okhotsk Ocean. *Tectonophysics* 269:113-129 DOI: 10.1016/S0040-1951(96)00140-0.
Zorin YA, Belichenko VG, Turutanov EK, Mazukabzov AM, Sklyarov EV & Mordvinova VV. 1995. The East Siberia transect. *International Geology Review* 37:154-175 DOI: 10.1080/00206819509465398.
